# Pegylated Arginine Deiminase Downregulates Colitis in Murine Models

**DOI:** 10.1155/2012/813892

**Published:** 2012-01-17

**Authors:** Helieh S. Oz, Jian Zhong, Willem J. S. de Villiers

**Affiliations:** ^1^Department of Physiology, College of Medicine, University of Kentucky Medical Center, Lexington, KY 40515-0298, USA; ^2^Department of Internal Medicine, College of Medicine, University of Kentucky Medical Center, Lexington, KY 40515-0298, USA; ^3^Division of Digestive Diseases and Nutrition, Department of Internal Medicine, College of Medicine, University of Kentucky Medical Center, Lexington, KY 40515-0298, USA

## Abstract

Arginine deiminase (ADI), an arginine-metabolizing enzyme involved in cell signaling, is dysregulated in multiple inflammatory diseases and cancers. We hypothesized that pegylated ADI (ADI-PEG) provide protection against colitis. *Methods*. Dextran sodium sulfate colitis was induced in IL-10-deficient and BALB/c (WT) mice. ADI-PEG was administered *i.p.*, and inflammatory mediators and pathology were evaluated. *Results*. Acute colitis in mice was manifested by increases in inflammatory biomarkers, such as serum amyloid A (SAA, *P* < 0.001), IL-12 p40, and disease index (3-Fold). In contrast, ADI-PEG significantly decreased clinical disease index, SAA levels, and inflammatory cytokines in blood as well as in colonic explants. Animals developed moderate (2.2 ± 0.3 WT) to severe (3.6 ± 0.5 IL-10 deficient) colonic pathology; and ADI-PEG treatment significantly improved the severity of colitis (*P* < 0.05). Marked infiltration of CD68^+^ macrophages and iNOS expression were detected in colonic submucosa in colitic animals but not detected in ADI-PEG-treated animals. *Conclusion*. ADI-PEG attenuated inflammatory responses by suppression of macrophage infiltration and iNOS expression in colitic animals. ADI-PEG can serve as a potential therapeutic value in IBD.

## 1. Introduction

Arginine deiminase (ADI) plays important role in cell signaling pathways, including apoptosis, differentiation, and transcriptional regulation [1, 2]. ADI is an arginine-degrading enzyme that catalyzes the hydrolysis of peptidylarginine to form peptidylcitrulline. ADI activity is dysregulated in multiple human diseases including rheumatoid arthritis (RA), inflammatory bowel disease (IBD), and cancer. Since, tumor cell lines require arginine for growth, selective elimination of arginine from the circulation has been suggested as a potential cancer treatment modality and currently in use in human trials [3]. Conversely, ADI inhibition with cl-amidine has been shown to reduce disease severity in the collagen-induced arthritis model of RA [4, 5].

Nitric oxide (NO) is known to be elevated in the colonic tissues of patients with active IBD and increased NO production and nitric oxide synthase (NOS) activity are detected in cultured mucosal explants from patients with active IBD, both ulcerative colitis and Crohn's disease [6–9]. Immunohistochemical analysis has identified inducible nitric oxide synthase (iNOS), a key inflammatory mediator, responsible for generation of NO and as the NOS isoform elevated in IBD [10, 11]. This increase-associated iNOS is localized to macrophages and epithelial cells, particularly in luminal epithelia that expose colonic tissue [11].

**Scheme 1:**
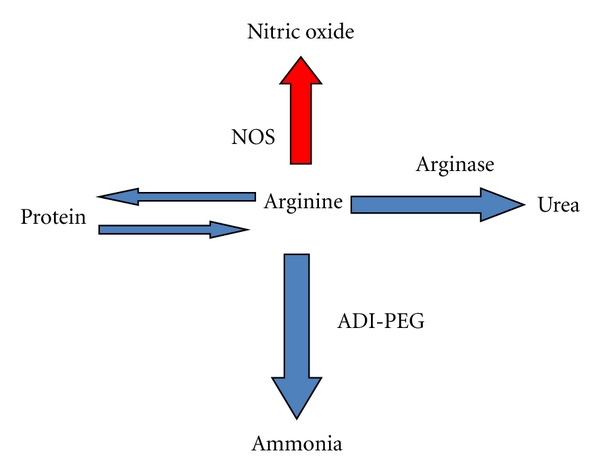
Arginine metabolism.

Additionally, the expression of iNOS in circulating monocytes and the percentage of iNOS-positive monocytes are increased in patients with active IBD as compared to healthy controls [12]. Arginine metabolism is likely to be another factor with regard to the roles of NOS in colitis. In addition to serving as a NOS substrate, arginine is a mediator for the enzyme arginase, which synthesizes ornithine, an intermediate in polyamine biosynthesis. Polyamines have been suggested to protect against colitis, possibly by stimulating reepithelialization and increasing arginase activity associated with healing following experimental colonic anastomosis [13, 14]. Arginine, is a nonessential amino acid, synthesized in normal cells and tissues from citrulline in a 2-step process using the urea cycle enzymes argininosuccinate synthase and argininosuccinate lyase. As an arginine metabolizing enzyme, ADI (Scheme 1), has major properties with respect to the issues of harmful and helpful NO and alternative arginine metabolism with preventing harmful NO generation [15].

In fact, the native ADI is very immunogenic with a short circulating lifespan of only 5 h. ADI covalently binds to polyethylene glycol (PEG) with molecular weight of 20 kDa and forms ADI-PEG. ADI-PEG is less antigenic with a significant increased circulating life span of about 5 days and reported to be safe in mice as well as humans [3, 16].

Although, ADI plays an important role in regulating cell signaling, the mechanism by which the enzyme's activity is regulated under physiological as well as pathological conditions leading to IBD is not fully discovered. Here we hypothesized the potential protective action of a microbial enzyme, ADI-PEG treatment against acute colitis.

## 2. Methods

Pegylated arginine deiminase (ADI-PEG) was prepared and provided by Dr. Steiner (University of KY; USA). Briefly, nonmammalian ADI gene from *Mycoplasma hominis *was expressed in *E. coli *and purified by anion-exchange chromatography with a POROS HQ resin column. The recombinant ADI was then pegylated according to the manufacture recommendation, and the enzyme activity was measured [16].

### 2.1. Animals

This study was approved and performed in accordance with the guidelines for the care and use of laboratory animals at the accredited University of Kentucky and Veterans Administration (VA) Medical Center in Lexington, KY, USA. IL-10-deficient mice were bred in animal facility at VA Medical Center and UK Medical Center. Male pups were weaned and at 5 weeks of age enrolled in this study.

In addition, five-week-old male BALB/c wildtype mice were purchased from Harlan Laboratories (Indianapolis, IN) and acclimatized for 10 days prior to the experiment. The IL-10-deficient mice were cohoused with wildtype mice (WT) in conventional condition, in microisolator cages, with free access to water and food (Harlan Teklad Laboratory Diet, Madison, WI) and kept in a room with a 12 h light/dark cycle.

### 2.2. Colitis Induction

 Colitis was induced in IL-10-deficient-and WT mice by oral ingestion of 3% dextran sodium sulfate (DSS) for 7 days and assessed by clinical disease index, inflammatory mediators, and histological grading scores. ADI-PEG low dose (5 IU) and high dose (10 IU) were chosen based on the safe and effective doses detected in previous cell culture studies. ADI-PEG was administered *i.p.* on day 0 and day 5 to the treated groups as ADI-PEG has a 1/2 lifespan of about 5-6 days.

### 2.3. The Clinical Disease Index

The clinical disease activity score was determined by the mean measurement of animal survival, extent of weight loss, stool consistency, presence of blood in the stool, anemia as expressed by the hematocrit, colonic weight and length, and prolapse collectively which were scored from 0, to present normal up to 10, the most severe case.

### 2.4. Colonic Histopathology

Colonic tissues were flushed with ice-cold phosphate-buffered saline (PBS pH 7.2) and cut longitudinally in half. One portion of each colon was Swiss-rolled and fixed in 10% formalin for histological examination. The remainder was snap-frozen in liquid nitrogen and stored at −80°C. The formalin fixed sections were processed and stained with hematoxylin and eosin (H and E) and evaluated under light microscopy. Severity of colitis was assessed with a histological semiquantitative grading score [17] and in a blinded manner. The scores were based on histological features with a numeric value (0 to 4) assigned according to the tissue involvement and severity of lesions that corresponded to either of the following criteria:

grade (0): No detectable lesions, no inflammatory cells, and normal mucosal appearance,
grade (1): Focal inflammatory infiltrate in the mucosa (25% involved),
grade (2): Mild multi-focal inflammation with moderate expansion to the mucosa (50% involved),
grade (3): Moderate multifocal inflammation with moderate expansion of the mucosa (75% involved),
grade (4): Severe diffuse inflammation with crypt epithelium disruption and ulceration (over 75% involved).

### 2.5. Plasma Analysis for Inflammatory Biomarkers Concentrations

Blood was collected via right heart ventricle puncture in lightly heparinized syringes and kept on ice. Sera were separated by 5 min centrifugation at 5000 ×g and stored at −80°C prior to the analysis.

### 2.6. Colonic Explant Production of Cytokines

Proximal sections of cleansed colonic cuffs from each mouse were weighted and washed with sterile PBS and the RPMI 1640 culture medium. Each explant was then plated in 24-well plates and cultured in 1 mL of complete RPMI 1640 medium supplemented with 5% fetal bovine serum (FBS, BioWhittaker, Walkersville, MD, USA) for 24 h at 37°C. LPS (10 ng/mL) was added into duplicate wells to stimulate the cells. Culture supernatant was collected with centrifugation, and aliquots were stored at −80°C until analyzed for inflammatory markers.

### 2.7. Inflammatory Biomarkers

Cytokines were measured by ELISA kits and assayed according to the manufacture's recommendation. The concentrations of IL-6, IL-12p40, and TNF*α* were measured by ELISA kits obtained from R and D (Minneapolis, MN, USA), and SAA was analyzed by Kits from BioSource (Camarillo, CA, USA).

### 2.8. Immunohistochemical Assay (IHC)

Paraffin-embedded sections were cut, deparaffinized with hexane, rehydrated in alcohol baths, washed in PBS, microwaved in antigen retrieval solution (high pH; DAKO, Carpenteria, CA, USA) for 5 min, washed with PBS, incubated in 0.3% H_2_O_2_-methanol for 10 min, and then rinsed with water. Slides were incubated at room temperature for 1.5 h in a blocking solution consisting of normal serum, rinsed with PBS, and then incubated with primary antibody at 4°C overnight. Rabbit polyclonal IgG against CD68 and iNOS was obtained from Zymed-Invitrogen. Invitrogen (Carlsbad, CA, USA) and Millipore (Billerica, MA, USA) respectively. The “Elite ABC” kit was used with biotinylated secondary antibodies and biotin-conjugated horseradish peroxidase and then developed using a 3-3′-diaminobenzidine solution per manufacturer's instructions. The slides were subsequently counterstained with Methyl Green (Dako, Carpenteria, CA, USA). The CD68 (the macrophage specific-marker) staining for the quantification of macrophages and anti-iNOS (iNOS positive macrophage) for the assessment of iNOS expression was performed to evaluate macrophage infiltration and activation in colonic tissue [18]. 

### 2.9. Nitric Oxide (NO) Analysis

NO was assessed indirectly by the measurement of its breakdown products, nitrite plus nitrate [19], in a microtiter plate assay. Briefly, nitrate was reduced into nitrite by incubation with nitrate reductase, untreated NADPH was oxidized, and then total nitrite was colorimetrically measured following incubation with the Griess reagent. Griess reagent: 1% (w/v) ammonium chloride was adjusted to pH 8.0 with sodium borate, 1 : 1solution of 0.1% (w/v) *N*-(1-naphthyl) ethylenediamine dihydrochloride, and 1% (w/v) sulfanilamide, in 5% (v/v) phosphoric acid.

### 2.10. Statistical Analysis

Results are expressed as the mean ± SEM unless otherwise stated. Data was analyzed with ANOVA followed by appropriate post hoc test (Tukey compared all pairs) using GraphPad Instat version 3 for Windows (San Diego, CA, USA). Statistical significance was set at  *P* < 0.05.

## 3. Results

Colitis was induced in WT and IL-10-deficient mice by oral ingestion of DSS in daily drinking water for 7 days. ADI-PEG was administered *i.p*. on day 0 and day 5 to the treated groups (ADI-PEG circulating 1/2 life is 5 days). Clinical disease index was determined by using parameters such as survival, extent of weight loss, the presence or absence of diarrhea, rectal bleeding, anemia, and prolapse.

**Figure 1:**
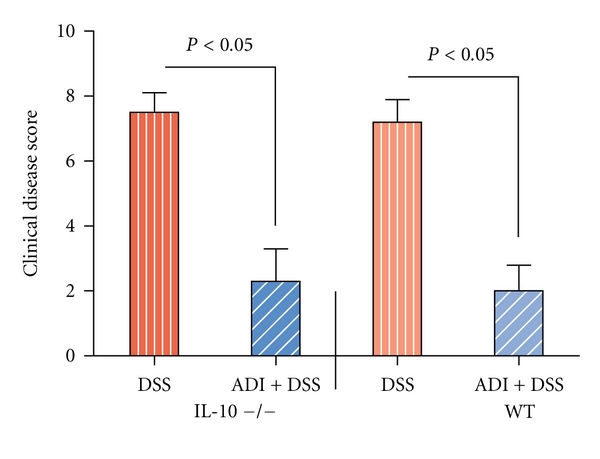
Effects of ADI-PEG therapy on the clinical disease index in DSS-induced acute colitis. ADI-PEG was administered *i.p* on day 0 and day 5. Clinical disease index was determined by parameters as means for extent of weight loss, presence of diarrhea, rectal bleeding, prolapse, and anemia (*n* = 6/group). ADI-PEG markedly decreased the clinical disease index in WT and IL-10-deficient colitic mice.

**Figure 2:**
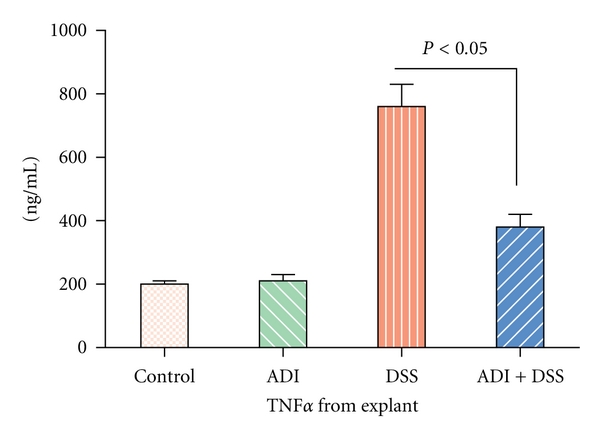
ADI-PEG treatment significantly decreased inflammatory response to LPS-stimulated TNF*α* production by colonic explants (*P* < 0.05).

DSS-exposed IL-10-deficient and WT animals all lost weight and developed anemia due to blood loss. In contrast, ADI-PEG treatment significantly protected DSS-exposed mice from weight loss and rectal bleeding compared to untreated DSS colitic animals (data shown collectively as disease index Figure 1). Over 80% of animals in both IL-10-deficient and WT groups developed diarrhea and occult blood, whereas only IL-10-deficient colitic animals progressed to prolapse. In contrast, ADI-PEG treatment significantly protected WT as well as IL-10-deficient animals against diarrhea, occult blood, and prolapse (Figure 1). Colitic animals developed significant increases at the levels of inflammatory cytokines including plasma concentrations of IL-6 and IL-12p40 (Tables 1 and 2). Additionally, marked increases in the production of TNF*α* (Figure 2), IL-6, and IL-12p40 were noted in the stimulated colonic explants (Tables 1 and 2) corresponding to inflammatory response in colitic mice both IL-10 deficient and WTs. In contrast, ADI-PEG-treated animals had a significant reduction of these inflammatory cytokines (*P* < 0.05) in their stimulated colonic explants (Tables 1 and 2 **)**.

Acute inflammatory biomarker, serum amyloid A (SAA), was significantly increased in the colitic IL-10-deficient (400-fold) and WT (30-fold) animals, while, ADI-PEG-treated animals exhibited significant reduction at the levels of SAA biomarker compared to untreated colitic animals (Tables 1 and 2). In addition, there was a significant increase at the plasma levels of NO production presumably released mainly from activated macrophages/monocytes. NO production was measured as the concentration of NO breakdown products (nitrate + nitrite) and was increased 2-fold in the colitic IL-10-deficient animals as compared to normal controls. Contrary to IL-10-deficient animals, the excess NO release did not reach statistical significance in WT animals as colitis was less severe in WT mice compared to IL-10-deficient counterparts. ADI-PEG treatment significantly ameliorated the NO release in colitic animals (Tables 1 and 2).

### 3.1. Colonic Histopathology

**Figure 3:**
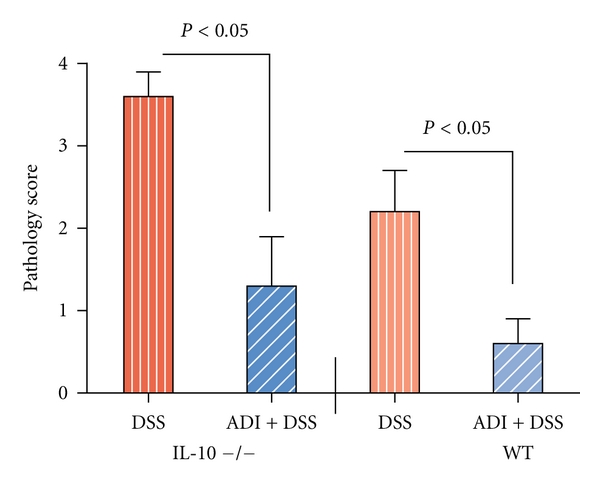
Effect of ADI-PEG on histopathology scores (0–4) according to the severity of lesions in DSS-colitic animals. Histopathological assessment revealed that ADI-PEG markedly attenuated DSS-induced colitis in WT as well as IL-10 deficient mice. (*n* = 6/group).

**Figure 4:**
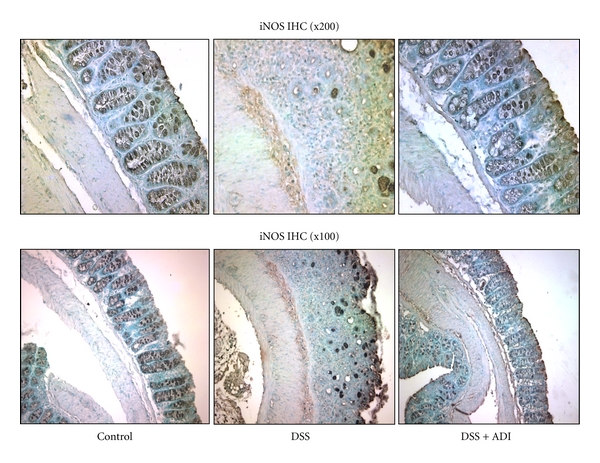
Effect of ADI-PEG on macrophage infiltration in colonic tissue from colitic IL-10-deficient mice. ADI-PEG markedly decreased submucosal macrophage infiltration and iNOS expression in DSS-induced colitic IL-10-deficient as well as WT mice (not shown). (*n* = 6/group).

DSS administration induced moderate colitis (2.2 ± 0.3) in WT mice to severe cases (3.6 ± 0.5) in IL-10-deficient mice (Figure 3) possibly due to more severe immune reaction to overgrowth of gut microbial due to the lack of protective IL-10 and overactivation of macrophages and inflammatory cells. This was in accordance with pathological manifestations including shortening of crypts, loss of brush boarder epithelial cells, thickening of submucosa, and ulcer formation in colitic animals as confirmed in histological sections (Figure 3). In general, the H and E histological damage scores were consistent with infiltration of inflammatory cells, extensive macrophage proliferation, followed by shortening of colonic length, and increased disease index in the colitic WT as well as IL-10-deficient animals. Furthermore, marked CD68^+^ macrophage infiltration in the submucosa of the colonic tissue was confirmed by immunohistochemical analysis in WT and IL-10-deficient colitic animals. Similar to CD68^+^ specific macrophage infiltration, the expression of macrophage derived iNOS was upregulated in the colonic macrophages (Figure 4) as well as local gut epithelia, supporting the notion that ADI-PEG provides protection against colitis by preventing CD68^+^  macrophage proliferation and iNOS activation in colonic tissues from IL-10 deficient and to a lesser extent in WT animals (data not shown).

Therefore, ADI-PEG-treated animals developed significantly less severe colitis as manifested by improved disease index (survival and increased body weight), decreased levels of inflammatory biomarkers, and decreased distal and proximal colonic histological damage scores. Colonic explants from ADI-PEG-treated mice released significantly less inflammatory cytokines compared with untreated DSS-colitic mice. Collectively, ADI-PEG was better tolerated in low dose and was more effective in normalizing SAA, plasma IL-6, and NO compared to high dose against colitis in animals (Table 1).

## 4. Discussion

These data demonstrate that ADI formulated with polyethylene glycol (ADI-PEG) effectively attenuated colonic inflammatory response induced in 2 phenotypic distinct IBD murine models. As anticipated in DSS-induced colitis model, the levels of the acute phase protein, SAA, are significantly elevated due to inflammatory cascade as occurs in colitic animals [17, 18, 20]. ADI-PEG markedly ameliorated the elevated levels of inflammatory mediators including SAA, IL-6, and IL-12p40, in sera as well as in colonic explants' supernatants from treated animals. IL-12p40 is specifically released by the activated macrophages involved in Th-1 mediated immune responses.

Macrophage infiltration and activation are one of the major potential mechanisms of DSS-induced colitis in animals presumably due to the overgrowth of gram-negative bacteria and are regulated by the luminal microbiota [21]. As CD68^+^-macrophage-specific marker and the iNOS activity in the colonic tissues were significantly elevated following inflammatory cascades by infiltration and activation of macrophages in both WT as well as IL-10-deficient colitic animals. Our data indicate that pegylated ADI effectively attenuates intestinal inflammation induced in colitis and diminishes macrophage proliferation and activation in these colitic animals.

The luminal NO level is shown to be increased in IBD patients [7, 8], and the plasma NO is elevated in active ulcerative colitis and Crohn's disease versus inactive state or normal controls [9]. Additionally, gene expression (mRNA) of inducible nitric oxide synthase, iNOS, is elevated in Crohn's and ulcerative colitis patients [22]. In addition, 2-fold increases at the plasma levels of NO production were presumably released from activated macrophages/monocytes in the colitic IL-10-deficient animals as compared to the normal controls. In contrast, the excess NO release did not reach statistical significance in WT animals, as colitis was less severe in WT mice compared to IL-10-deficient counterparts. In this study, the reduction in the colonic inflammation is possibly associated with decreased macrophage-derived NO release by ADI-PEG, as demonstrated by a significant decrease in macrophage infiltration and iNOS expression in WTs as well as IL-10 deficient mice. Further, nNOS appears to protect against DSS-induced colitis, since disease is more severe in nNOS−/− mice as compared to WT animals [23]. These findings indicate that ADI-PEG is a promising agent for the elucidation of the role of arginine-related events in the pathogenesis of colitis.

However, there is controversy with regard to the role of NO in IBD since NO has been shown to have some protective role, indicating reduction in double IL-10/iNOS−/− mice [24] and some beneficial effect with an NO donor [25]. Dissimilarity in experimental design may contribute to some of these variations [26]. Also, that NO may play dual function, both deleterious and protective function in the gut [27]. It is anticipated that inflammation-related NO synthesized by macrophage iNOS is highly pathogenic, while intestinal epithelial iNOS may synthesize protective NO in nature and function in healing and reepithelialization [28, 29]. In addition, increased arginase activity is observed in IBD [30]. Arginine metabolism is likely to be another factor with regard to the roles of NOS in colitis. Macrophage arginase expression is stimulated by lipopolysaccharides, LPS, IL-10, alone or in combination to (LPS + IL-10), a protective cytokine in IBD, which synergizes with respect to increased arginine expression [31]. Indeed, as proposed previously “more global” considerations of arginine metabolism, particularly arginase and NOS, might be the key elements to reveal the roles of NO in IBD [32] and further the potential mechanism of ADI-PEG on the development of IBD.

In the current study, the microbial enzyme, *Mycoplasma hominis *ADI gene was cloned and expressed in *Escherichia coli* and then conjugated with PEG to increase the circulating half-life from 5 h to 5 days and to drastically decrease the immunogenicity of the recombinant enzyme [16]. Generally, ADI converts plasma arginine into citrulline, a metabolite which is taken up and converted back into arginine only by normal cells and tissues but not by tumor cells such as hepatocellular carcinoma [33]. The reduction in the intestinal inflammation is related to decreased inflammatory cytokines as well as decreased macrophage-derived NO levels by ADI-PEG, as shown by reduced macrophage infiltration and colonic iNOS expression.

Overall, DSS-induced colitis was more severe in IL-10 deficient mice than in WT animals as IL-10 cytokine plays an important role as an anti-inflammatory element with a protective action against colitis [20]. Also, that ADI-PEG acts through the inhibition of macrophage activation and proliferation.

 Overall, ADI-PEG significantly ameliorated colitic symptoms and pathology in treated animals by blocking infiltration/proliferation of these inflammatory cells in tissues. Therefore, ADI-PEG is a possible promising agent against colitis as well as for the elucidation of the role of arginine-related events in the pathogenesis of colitis. 

## 5. Conclusion

ADI-PEG treatment protects against colitis in 2 distinct phenotypic animal models, BALB/c wildtypes as well as IL-10 deficient mice, presumably due to the attenuation of inflammatory markers such as SAA, suppression of macrophage infiltration, and iNOS expression in colonic tissues. As such, ADI-PEG can serve as a potential therapeutic value in IBD.

## Figures and Tables

**Table 1:** IL-10 deficient-mice: comparison of inflammatory markers in plasma and colonic explants in IL-10-deficient (IL-10−/−) mice treated with DSS and ADI-PEG versus sham controls. *P < 0.05, **P < 0.01, and  ***P < 0.001.

	Plasma				Colonic explant + LPS	
Inflammatory markers	IL-6 (pg/mL)	IL-12p40 (pg/mL)	SAA (*μ*g/mL)	NO (*μ*M)	IL-6 (ng/mL)	IL-12p40 (pg/mL)
Control	24 ± 3	233 ± 18	3 ± 1	80 ± 5	126 ± 54	170 ± 56
ADI	25 ± 4	297 ± 13	38 ± 31	50 ± 5	179 ± 83	199 ± 113
DSS	202 ± 28**	319 ± 20	1591 ± 550***	205 ± 39*	1768 ± 629***	1194 + 130**
DSS + ADI-PEG (low dose)	49 ± 15	292 ± 23	276 ± 130**	60 ± 2	257 ± 73*	255 ± 28*
DSS + ADI-PEG (high dose)	126 ± 20*	295 ± 7	1105 ± 662*	90 ± 20	108 ± 41**	313 ± 85

**Table 2:** WT mice: comparison of inflammatory markers in plasma and colonic explants in BALB/c (WT) mice treated with DSS and ADI-PEG versus sham controls. *P < 0.05, **P < 0.01, and  ***P < 0.001.

	Plasma			Colonic explant + LPS
Inflammatory markers	IL-6 (pg/mL)	SAA (*μ*g/mL)	NO (*μ*M)	IL-12p40 (pg/mL)
Control	45 ± 14	12 ± 1	24 ± 1	550 ± 25
ADI	27 ± 5	48 ± 37	27 ± 2.5	650 ± 50
DSS	182 ± 27*	427 + 121**	36 ± 3	875 ± 150*
DSS + ADI-PEG (Low dose)	55 ± 9	24 + 12*	24 ± 3	475 ± 50

## Abbreviations

ADI: Arginine deiminase
CD: Crohn's disease
DSS: Dextran sodium sulfate
IBD: Inflammatory bowel disease
IHA: Immunohistochemistry
iNOS: Inducible nitric oxide synthase
IU: International unit.
LPS: Lipopolysaccharide
MΦ: Macrophage
PEG: Polyethylene glycol
SAA: Serum amyloid A
TNF*α*: Tumor necrotic factor
UC: Ulcerative colitis
WT: Wildtype
IL-10: Interleukin 10.
